# Association of a Human *FABP1* Gene Promoter Region Polymorphism with Altered Serum Triglyceride Levels

**DOI:** 10.1371/journal.pone.0139417

**Published:** 2015-10-06

**Authors:** Xian-E Peng, Yun-Li Wu, Yi-bing Zhu, Rong-dong Huang, Qing-Qing Lu, Xu Lin

**Affiliations:** 1 Key Laboratory of Ministry of Education for Gastrointestinal Cancer, School of Basic Medical Sciences, Fujian Medical University, Fuzhou, China; 2 Department of Epidemiology and Health Statistics, School of Public Health, Fujian Medical University, Fuzhou, China; 3 Fujian Key Laboratory of Tumor Microbiology, Department of Medical Microbiology, Fujian Medical University, Fuzhou, China; 4 Department of Gastroenterology, Union Hospital of Fujian Medical University, Fuzhou, China; University of Basque Country, SPAIN

## Abstract

Liver fatty acid-binding protein (L-FABP), also known as fatty acid-binding protein 1 (FABP1), is a key regulator of hepatic lipid metabolism. Elevated FABP1 levels are associated with an increased risk of cardiovascular disease (CVD) and metabolic syndromes. In this study, we examine the association of *FABP1* gene promoter variants with serum FABP1 and lipid levels in a Chinese population. Four promoter single-nucleotide polymorphisms (SNPs) of *FABP1* gene were genotyped in a cross-sectional survey of healthy volunteers (*n* = 1,182) from Fuzhou city of China. Results showed that only the rs2919872 G>A variant was significantly associated with serum TG concentration(P = 0.032).Compared with the rs2919872 G allele, rs2919872 A allele contributed significantly to reduced serum TG concentration, and this allele dramatically decreased the *FABP1* promoter activity(*P* < 0.05). The rs2919872 A allele carriers had considerably lower serum FABP1 levels than G allele carriers (*P* < 0.01). In the multivariable linear regression analysis, the rs2919872 A allele was negatively associated with serum FABP1 levels (***β = —***0.320, *P* = 0.003), while serum TG levels were positively associated with serum FABP1 levels (***β =*** 0.487, *P* = 0.014). Our data suggest that compared with the rs2919872 G allele, the rs2919872 A allele reduces the transcriptional activity of *FABP1* promoter, and thereby may link *FABP1* gene variation to TG level in humans.

## Introduction

Clinical lipid disorders are associated with enormous public health significance and increasing societal burden in many countries [1]. Epidemiological evidence supporting raised plasma triglycerides (TG) is emerging as an independent risk factor for Type 2 diabetes, fatty liver, metabolic syndrome and atherosclerotic cardiovascular disease (CVD) [2,3,4]. The concentration of TG in an individual depends on the interplay between genetic and environmental factors. Although environmental factors such as smoking and alcohol consumption are triggers, hypertriglyceridemia has a tendency to cluster within families, suggesting that genetic factors also contribute to the risk of developing this disorder. Although many genetic candidates have been discovered to date, these only explain a small fraction of the total inter-individual variation in plasma TG levels [5,6,7]. Therefore, the search for the genetic factors that explain the increased susceptibility to hypertriglyceridemia is a current focus of research.

The Liver fatty acid-binding protein (L-FABP), also known as fatty acid-binding protein 1 (FABP1), is a member of the FABP family that is found in abundance in the cytosol of liver parenchymal cells [8]. It serves as an intracellular acceptor of long-chain fatty acids (LCFA), following their cellular uptake, trafficking, and mitochondrial oxidation [9,10]. Studies performed in vitro and in vivo indicate that FABP1 plays a role in the incorporation of fatty acids into TGs [11]. For example, murine FABP1 overexpression increased LCFA uptake and increased hepatic TG, while decreased LCFA uptake and reduced hepatic TG levels have been found in *FABP1* knockout mice [12,13,14]. Given the key role of FABP1 in lipid metabolism, it is conceivable that variation in the *FABP1* gene, either in the coding region or regions that regulate *FABP1* expression, could directly influence plasma TG levels or others lipid-related phenotypes. In fact, a highly conserved c.340A>G missense mutation in exon 3 of the human *FABP1* gene alters a threonine (T) to alanine (A) at position 94 (T94A) and is thought to contribute negatively to FA binding. This variant is associated with increased serum triglycerides and LDL cholesterol levels [15], reduced response to lipid-lowering therapy with fenofibrate [16], as well as the development of nonalcoholic fatty liver disease (NAFLD)[17]. However, little is known about the association of polymorphisms in the promoter region of the *FABP1*gene with lipid metabolism.

In the present study, common mutations in the promoter of the *FABP1* gene responsible for alterations in serum TG levels was identified. Furthermore, the association of serum TG levels with altered FABP1 levels attributed to this mutation in the promoter region was analyzed.

## Materials and Methods

### Subjects

#### Population Selection

The association between the promoter polymorphism and the risk for dyslipidemia in the Han population was assessed using a cross-sectional survey. A total of 1,182 subjects (male/female: 817/365, aged 18–72 years) were recruited from among individuals visiting the Union Hospital of Fujian Medical University for regular medical check-ups between August 2012 and January 2013. Participants were selected according to the following criteria, 1) absence of prior CVD, severe acute disease, Type 1 diabetes or pregnancy; 2) had not received lipid-lowering treatment or any other drug-modifying lipid measures. In addition, secondary causes of lipid disorders were excluded in all subjects based on clinical history and blood tests. All subjects enrolled were of Chinese Han ethnicity. This study was conducted in accordance with the guidelines of the 1975 Declaration of Helsinki and was approved by the Ethics Committee of the Fujian Medical University. Each subject gave written informed consent before participation in the study.

### Data Collection

#### Interview

A pilot-tested structured questionnaire was administered by trained interviewers to collect information on demographic characteristics (e.g. age, education level, job, marital status, sex), as well as relevant information on smoking habits, tea and alcohol drinking habits, physical exercise, personal medical history, and potential others risk factors. Individuals reporting regular tobacco use in the previous 6 months were classified as smokers. Alcohol and tea drinkers were defined as those who had drunk alcohol/tea at least once per week for more than 6 months. Physical activity was ascertained by asking participants about their workplace physical activity and how often in the previous month they had engaged in any number of different activities, including walking, jogging, cycling and swimming. The participants were grouped into two broad categories based on their total score: inactive (lower physical activity) and active (higher physical activity).

#### Anthropometric Measurements and Laboratory Evaluation

Weight and standing height were measured in a standardized fashion by a trained examiner. Body mass index (BMI) was calculated as the ratio of weight (kilograms) to the square of height (meters). Systolic and diastolic blood pressure was determined in two consecutive measurements on the upper arm using an automated sphygmomanometer (HEM- 907, Omron, Kyoto, Japan) with subjects in a seated, resting position; The mean of blood pressure was used for analysis. An overnight fast venous blood samples were drawn after an anthropometric examination. The levels of total cholesterol (TC), high-density lipoprotein cholesterol (HDL-C) and low density lipoprotein cholesterol (LDL-C), triglycerides (TGs), fasting plasma glucose (FPG), and uric acid (UA), in addition to liver function tests, were measured by standard clinical laboratory techniques, which have been described previously [17].

### Genotype Analysis

The genomic region harboring *FABP1* promoter was examined to select single nucleotide polymorphisms (SNPs) based on the linkage disequilibrium (LD) patterns of Chinese Han population in Beijing (CHB) from Hapmap database. Then we used the pairwise tagging method of the Haploview v4.2 software (Broad institute, Cambridge, MA, USA) to capture SNPs with a minimum minor allele frequency (MAF) of >0.05 and a minimum r^2^ of >0.8. As result, four SNPs (rs2919872, rs2970901, rs2970902, and rs2970903) were selected from unrelated Han Chinese individuals in Beijing. Finally, widely used software for TF binding site prediction MatInspector and CHIP Mapper[18], were used to predict possible transcription changes due to the presence of a variant.

DNA was isolated from EDTA-treated blood samples following standard procedures. These polymorphisms were genotyped by iPlex technology based on a MassARRAY platform in all subjects. Primers for the amplification and extension reactions were designed using the Mass Array Assay Design Version 3.1 software (Sequenom, San Diego, CA, USA), and SNP genotypes were determined according to the iPLEX protocol provided by the manufacturer. Genotyping assays were performed by laboratory personnel who were blinded to the lipid status of each subject. The genotyping quality was examined in a detailed QC procedure consisting of a >95% successful call rate, duplicate calling of genotypes, internal positive control samples and Hardy–Weinberg Equilibrium (HWE) testing.

### FABP1 Measurement

Serum FABP1 levels were measured in a subset of subjects with different FABP1 rs2919872 genotypes. Subjects with impaired glucose metabolism (indicated by an oral glucose tolerance test), abnormal liver function, a history of smoking, alcohol drinking, or tea drinking were excluded. There were no significant differences in the age and sex distributions of the subjects in this subset compared with those of the total subject population. All evaluations were performed on an aliquot of serum collected after overnight fasting and stored at −80°C. Serum FABP1 levels were evaluated in 95 subjects with different rs2919872 genotypes using the commercially available FABP1 ELISA kit (GWB-HFABP1, GeneWay Biotech, San Diego, CA, USA) according to the manufacturer’s instructions.

### *FABP1* Promoter Luciferase Reporter Constructs

The pGL3-rs2919872G plasmid harboring the human *FABP1* promoter with the rs2919872 G allele was constructed by ligation of the PCR-generated full length human *FABP1* promoter (nucleotides –2125 to +50, relative to the transcription start site) into the pGL3-Basic luciferase reporter plasmid (Promega, Madison, WI, USA), as described previously in our study (also designated pGL3B–2125) [19]. Fusion PCR was performed to create the rs2919872 A allele of the FABP1 promoter (designated pGL3- rs2919872A) using pGL3-rs2919872G as a template. Briefly, two pairs of primers, i.e., P1/P3 and P2/P4, were used to amplify the fragments flanking the site of rs2919872A. The two amplified fragments were annealed and fused by PCR with P1 and P4. The fused fragment was digested with *Tth*111I and *Bst*AP1and used to replace the *Tth*111I-*Bst*AP1 fragment of pGL3-rs2919872G. The amplified sequences were confirmed by DNA sequencing. The primers used were: P1 (forward): 5′ TGCCCGCTGTTCAGGTAGTC 3′, P2 (forward): 5′ TGAGGGGGTGCTTGTAAAGAGCTGCCTCAGAGGCAG 3′, P3 (reverse): 5′ TCTTTACAAGCACCCCCTCA 3′, P4 (reverse): 5′ ACAAGTGTGT GGGTGCATGT G 3′.

### Cell Culture and Transfection and Dual-luciferase Reporter Assay

The human hepatoblastoma cell line HepG2 (HB–8065, ATCC, VA, USA) and the hepatoma cell line Huh7 (JCRB0403, Japan) were maintained in Dulbecco’s modified Eagle medium (DMEM, Invitrogen) supplemented with 10% (v/v) fetal bovine serum (FBS, Invitrogen) at 37°C in a humidified atmosphere containing 5% CO_2_. Cells were seeded (2×10^5^ cells/well) in 12-well plates and transfected with the empty pGL3-Basic vector (a promoterless control) or with pGL3- rs2919872G or pGL3- rs2919872 A constructs using Lipofectamine 2000 (Invitrogen). The pRL-SV40 plasmid (Promega), containing *Renilla reniformis* luciferase, was cotransfected into cells as a normalizing control. For each plasmid, triplicate transfections were performed in three independent transfection experiments. After 48 h, intracellular luciferase activity was determined in a total of 20 μg of cell lysate using the Dual-Luciferase Reporter Assay System (Promega) according to the manufacturer’s recommendations. Luminescence measurement was carried out on a luminometer (Orion II Microplate Luminometer, Berthold Detection Systems, Germany). The relative luciferase units (RLU) were determined by comparison with the promoterless pGL3-Basic plasmid, which was assigned an arbitrary value of 1.

### Statistical Analysis

Coefficients of skewness and kurtosis were calculated to test deviation from a normal distribution. Logarithmic transformation was performed on the individual values of skewed variables, and a normal distribution of transformed values was confirmed before statistical analysis and significance testing. Unless otherwise stated, data are presented as means ± standard deviation (SD) for continuous variables and as frequencies or percentages for categorical variables. Differences in mean values were assessed using unpaired *t*-tests, one-way ANOVA or the Mann–Whitney U-test, as appropriate. Categorical variables were compared using *χ* ^*2*^ test. For each SNP, Hardy–Weinberg equilibrium (HWE) was checked in subjects using the *χ* ^*2*^ test. All tests were performed on the basis of an additive, dominant or recessive model. Previous studies have suggested that the multiple testing correction may hid many true-positive loci[20], and reduce the number of tests may be advantageous for finding true-positive loci [21,22]. Therefore, P-values were not adjusted for multiple tests in the present study.

In addition, multiple linear regression was performed to examine the independent predictors of serum TG or FABP1 concentrations with adjustment for age, sex, income, marital status, education, smoking, tea drinking, BMI and other clinical features. Before statistical analysis, raw TG and FABP1 level phenotype data were transformed into natural log (ln) values to overcome high-value deviation. All analyses were performed using the SPSS for Windows statistical package, Version 18.0 (SPSS). For all tests, *P*-values less than 0.05 were considered to indicate statistical significance.

## Results

### Subject Status and General Data

The study population included 817 males and 365 females, with a mean age of 45.06 ± 12.93 years (18–72 years). Clinical features, anthropometric variables and laboratory findings for all individuals are shown in Table 1. There were no significant differences in the distributions of LDL-C, AST, marital status, education or income between males and females included in the study (all *P* > 0.05). Compared with females, males showed significantly higher levels of BMI, SABP, DABP, ALT, and TG, and lower levels of TC, FPG, and HDL-C (all *P* < 0.05). In addition, the frequencies of active physical activity, smoker and alcohol drinkers were higher frequency among the male subjects than among the female subjects.

**Table 1:** Clinical characteristic of 1,182 participants.

Variables	All cases (*n* = 1182)	Men (*n* = 817)	Women (*n* = 365)	*P*
Age(years; mean ± SD)	45.06±12.93	43.56±13.36	48.42±11.22	<0.01
BMI(kg/m^2^; mean ± SD)	23.80±3.00	24.08±3.03	23.20±3.10	<0.01
SABP(mmHg; mean ± SD)	76.72±10.72	77.44±10.66	75.13±10.69	<0.01^c^
DABP(mmHg; mean ± SD)	124.27±13.17	125.14±12.98	122.33±13.40	<0.01^c^
AST (IU/L; mean ± SD)	24.58±25.94	25.56±30.32	22.38±10.77	.051^c^
ALT(IU/L; mean ± SD)	31.57±58.13	35.05±67.88	23.78±23.41	<0.01^c^
TC (mmol/L; mean ± SD)	5.23±1.08	5.18±1.06	5.34±1.11	.024^c^
TG (mmol/L; mean ± SD)	1.54±1.02	1.62±1.03	1.35±1.00	<0.01^c^
FPG (mmol/L; mean ± SD)	5.60±1.22	5.58±1.15	5.65±1.36	<0.01^c^
HLD-C (mmol/L; mean ± SD)	1.49±0.61	1.43±0.68	1.62±0.40	<0.01^c^
LDL-C (mmol/L; mean ± SD)	3.29±1.10	3.28±1.08	3.32±1.13	0.557^c^
Per capita incoming				>0.05^b^
Low, *n*(*%*)	314 (26.54)	192 (23.50)	122 (33.33)	
Middle, *n*(*%*)	486 (41.10)	312 (38.19)	174 (47.62)	
High, *n*(*%*)	383 (32.36)	313 (38.31)	70 (19.05)	
Marriage(married), *n*(*%*)	1017 (86.04)	663 (81.15)	354 (96.99)	>0.05^b^
Smoking status(smoking)	307 (26.00)	302 (36.96)	5 (1.45)	<0.01
Tea drinking(drinking)	589 (49.87)	436 (53.37)	153 (42.03)	
Alcohol drinking(drinking)	279 (23.64)	272 (33.29)	7 (2.04)	<0.01
Physical activity(active)	316(38.68)	105(28.77)		<0.01
Education, *n*(*%*)				>0.05^b^
≤6 years	548 (46.35)	405 (49.57)	143 (39.13)	
≤9 years	506 (42.80)	326 (39.90)	180 (49.28)	
>9 years	128 (10.86)	86 (10.53)	42 (11.59)	

Continuous variables are expressed as mean ± SD; Categorical variables are expressed as frequency (percent). *n*: number of individuals; SD: standard deviation.

*P*-values a: obtained by Student's *t*-test for normal distributed continuous variables

b: obtained by Pearson Chi square test for categorical variables

c: obtained by Mann-Whitney U-test for continuous variables deviation from a normal distribution.

### Analysis of the Association of Various Quantitative Phenotypes with the *FABP1* Gene Promoter SNPs

**Fig 1:**
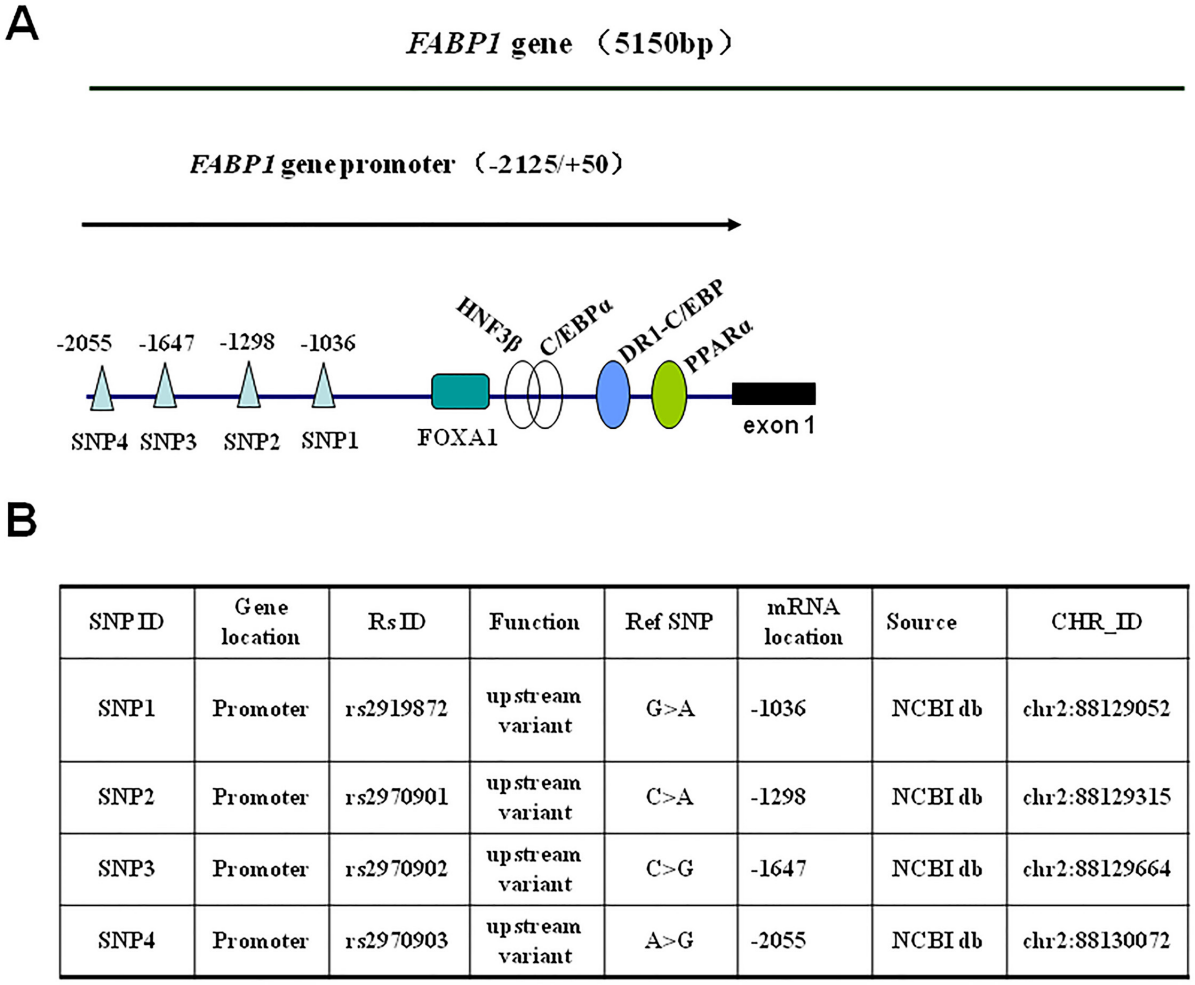
SNPs in the *FABP1* gene promoter. A. Schematic diagram showing the positions of SNPs and known binding sites for nuclear factors. Four SNPs (SNP1 to SNP4), located at -2,055 nt, -1,647 nt, -1,298 nt, and -1,036 nt respectively, were genotyped within the FABP1 promoter (-2,125 nt/+51 nt). The known binding sites for C/EBPα (CCAAT-element binding protein alpha), HNF3β (human nuclear factor beta), DR1-C/EBP (direct repeat with 1 base spacing/CCAAT-element binding protein), PPARα (peroxisome proliferator-activated receptor alpha) in the *FABP1* gene promoter have been reported previously [19,23]. B. SNP information. SNP1 to SNP4 are listed from 5′–3′ of the FABP1 promoter. “CHR-ID” indicates the chromosome ID of the four SNPs in the *FABP1* gene promoter. “RS ID”, reported in UCSC or NCBI website. “Function” indicates the altered genetically-coded function of the SNPs. “Ref SNP” indicates SNP form in the original reference sequence. “mRNA location” corresponding mRNA positions are shown for each SNP. NCBI db is available from http://www.ncbi.nlm.nih.gov/SNP.

The promoter *SNPs* information of *FABP1* gene was presented in Fig 1[19,23]. These SNPs were genotyped in all 1,182 individuals using iPlex technology based on a MassARRAY platform, and were commonly distributed in the study samples. The corresponding frequencies of the rs2970902G, rs2919872A, rs2970903A, and rs2970901A alleles among the participants were 0.160, 0.234, 0.053 and 0.232, respectively, and all of the tested SNPs were in Hardy–Weinberg equilibrium (*P* > 0.05).

Given that the involvement of FABP1 in lipid metabolism, we investigated the association of variants in the promoter sequence of this gene with altered serum lipid levels. The single allelic analysis showed that rs2919872 was associated with serum TG levels (*P* = 0.032, Table 2). In allele-specific phenotype analysis, the mean serum TG levels associated with rs2919872 G>A suggested that the A allele decreases the levels of serum TG, with AA homozygotes having a 14.47% decrease in serum TG levels (1.36 mmol/L vs. 1.59 mmol/L) relative to homozygotes for the GG allele and this effect was apparently recessive. We found no significant associations between rs2919872 and other lipid characteristics including TC, HDL-C and LDL-C. Among the other three tested polymorphisms (rs2970901, rs2970902, and rs2970903), no significant associations were identified with either serum TG levels or other clinical characters (data not shown). It was noteworthy that *FABP1* SNPs (rs2919871, rs1441644, rs2241883, rs2970902, rs2970903 and rs2919867) were previously reported not to be associated with lipid levels in the European population [24]. However, these SNPs are not in high linkage disequilibrium (LD) with the SNP rs2919872.

**Table 2:** Associations of the *FABP1* gene promoter variant rs2919872G>A with investigated parameters.

Quantitative phenotype	GG *(n =* 705)	GA (*n* = 401)	AA (*n* = 75)	*P* ^c^_add_	*P* ^d^_dom_	*P* ^e^_Recessiv_
Age (years) ^a^	44.77±12.61	45.49±13.31	45.31±14.15	0.667	0.372	0.855
SABP (mm Hg) ^b^	124.41±13.43	123.46±12.59	127.23±13.65	0.065	0.688	0.051
DABP (mm Hg) ^b^	76.82±10.44	76.14±11.02	78.94±11.55	0.103	0.728	0.061
BMI (kg/m^2^) ^a^	23.77±2.98	23.86±3.23	24.09±3.28	0.639	0.461	0.423
HDL-C (mg/dL) ^b^	1.49±0.54	1.49±0.76	1.48±0.34	0.993	1.000	0.911
LDL-C (mg/dL) ^b^	3.29±1.13	3.30±1.03	3.31±1.16	0.982	0.884	0.871
TC (mg/dL) ^b^	5.26±1.11	5.17±1.02	5.24±1.11	0.438	0.232	0.954
TG (mg/dL) ^b^	1.59±1.09	1.51±0.98	1.36±0.43	0.193	0.266	0.032
FPG (mg/dL) ^b^	5.53±0.82	5.72±1.74	5.66±1.07	0.057	0.211	0.637
ALT (IU/L) ^b^	30.22±33.28	34.75±90.03	28.73±22.89	0.425	0.312	0.648
AST (IU/L) ^b^	24.36±18.29	25.35±37.67	22.86±8.46	0.693	0.707	0.545

Continuous variables are expressed as mean ± standard deviation (SD); *n*: number of individuals

*P*-values a: obtained by one-way ANOVA

b: obtained by Kruskal–Wallis test or Mann-Whitney U-test

c additive model: GG vsGA. GG vs AA, and GA vs. AA

d:a dominant model: GG vs AA+GA

e: a recessive model (GA+GG vs.AA)

The effects of rs2919872 genotype on TG levels were further assessed using multiple linear regression models with adjustment for age, sex, income, marital status, education, smoking, tea drinking, BMI and other clinical features. TG values were defined as the dependent variable and were log-transformed before statistical analysis. In stepwise regression analysis, rs2919872G>A (recessive model) (β = -0.0515, P < 0.05), BMI (β = 0.134, P < 0.001), TC (β = 0.437, P < 0.001), HDL-C (β = -0.243, P < 0.001), LDL-C (β = -0.209, P < 0.001), SBP (β = 0.123, P < 0.001), and ALT (β = 0.063, P < 0.05), in this order, were independently associated with serum logTG concentrations (Table 3), explaining a total of 29.9% of its variability. The results were similar when age, sex, fasting glucose, income, marital status, education, smoking, alcohol drinking and tea drinking were added to the model.

**Table 3:** Variables independently associated with plasma TG levels by multivariate lineal regression analysis.

Variables	Standardized coefficient(*β*)	*P*	Adjusted R^2^
**Model 1** ^a^			
rs2919872G>A (recessive model)	-0.061	0.037	0.003
**Model 2** ^b^			0.299
rs2919872G>A (recessive model)	-0.051	0.042	
BMI	0.134	0.000	
TC	0.437	0.000	
HDL-C	-0.243	0.000	
UA	0.201	0.000	
LDL-C	0.209	0.000	
SABP	0.123	0.000	
ALT	0.063	0.011	
**Model 3** ^c^			0.303
rs2919872G>A (recessive model)	-0.049	0.046	
Others investigated characteristics			

a: Only the variable rs2919872G>A was entered into this model

b: Variables were selected by a forward stepwise selection procedure, in which variables were sequentially entered into this model.

c: All variables were entered into this model in a single step.

#### Functional Relevance of rs2919872

**Fig 2:**
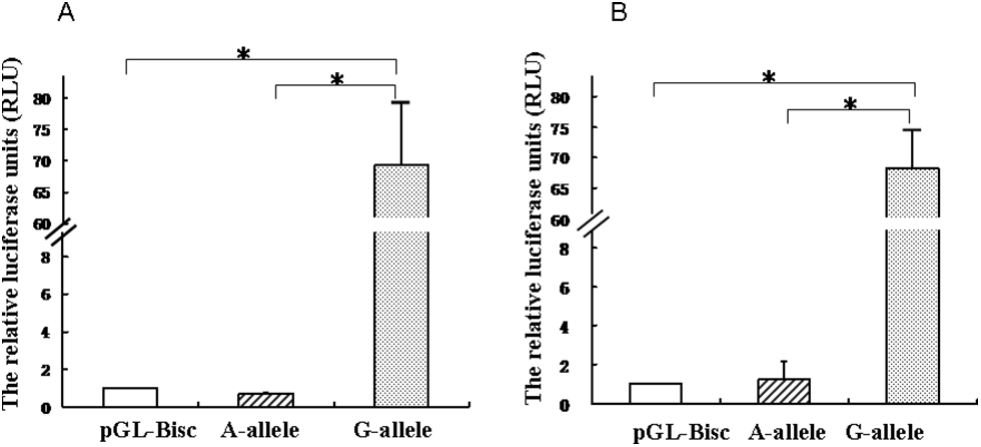
FABP1 promoter activities in A. HepG2 cells and B. Huh7 cells. Cells were co-transfected with 10 ng of the Renilla luciferase expression vector pRL-SV40 and 0.2 μg each of the pGL3-rs2919872G and pGL3-rs2919872A plasmids; the promoterless pGL3-Basic vector served as the negative control. Intracellular luciferase activity was measured 48 h after transfection. The relative luciferase units (RLU) were determined by comparison with the promoterless pGL3-Basic plasmid, which was assigned an arbitrary value of 1. Each transfection was performed in duplicate and the data are expressed as the mean ± SD of three separate experiments. (*P < 0.05).

Variations in regulatory sequences might influence gene expression following binding of transcriptional activators or inhibitors that instruct their regulatory control. Therefore, we examined the effect of the G>A polymorphism at rs2919872 on FABP1 promoter activity. To address this issue, the full length human FABP1 promoter (from −2125 to +51, relative to the transcription start site), with either the G or A at position rs2919872, was cloned into the pGL3-basic luciferase reporter plasmid. These two constructs, i.e., pGL3-rs2919872G and pGL3-rs2919872A, were then transfected separately into HepG2 and Huh7 hepatoma cells. As shown in Fig 2, cells transfected with pGL3-rs2919872G displayed much higher luciferase activity than that of cells transfected with pGL3-rs2919872A, suggesting that the rs2919872 A allele dramatically reduced FABP1 promoter activity.

#### Correlation of Serum FABP1 Concentration with the rs2919872 G>A Polymorphism in *FABP1* Promoter

**Fig 3:**
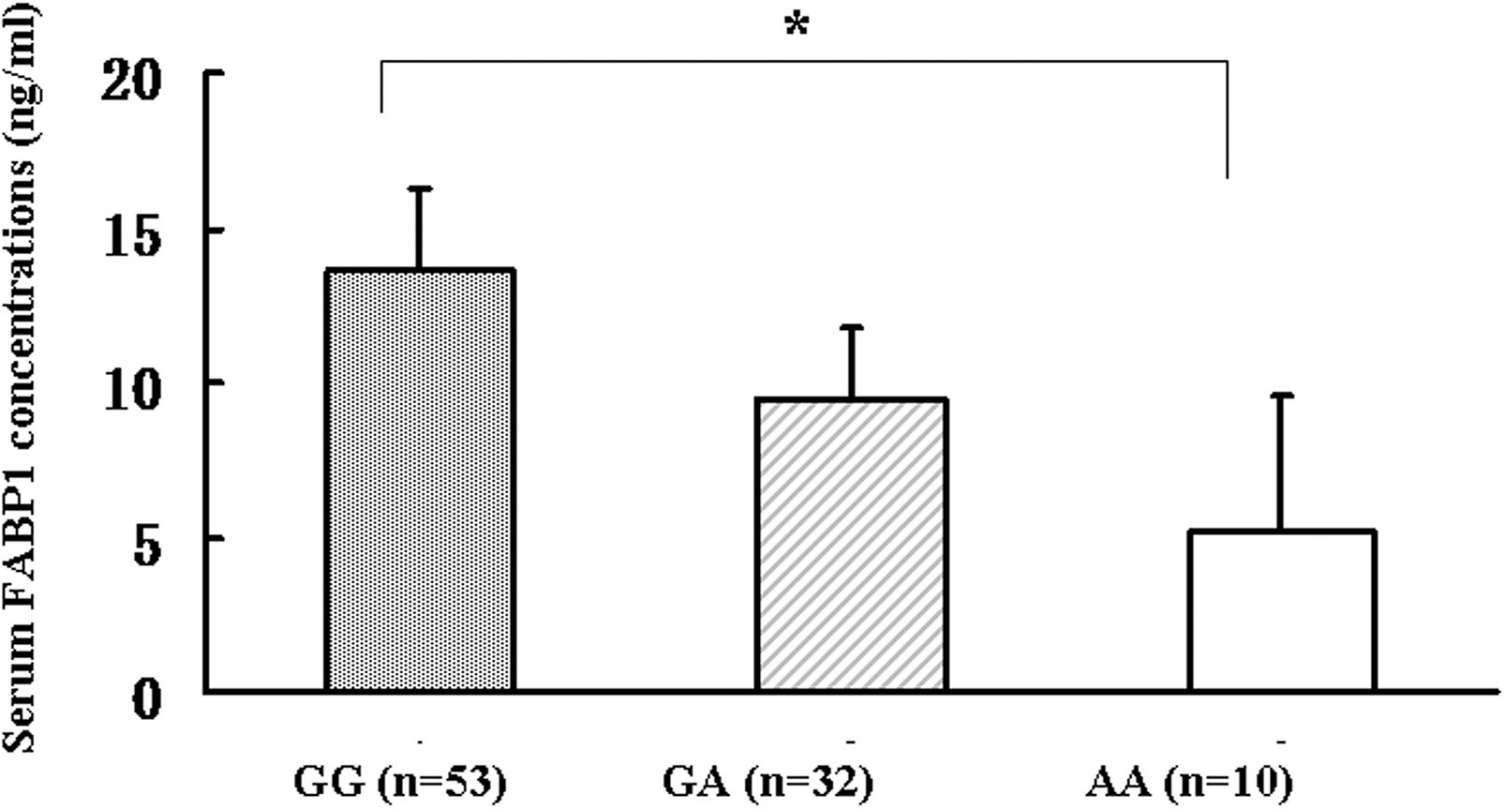
Serum FABP1 levels in healthy individuals with different *FABP1* rs2919872 genotypes. Serum FABP1 levels (geometric mean ± geometric standard deviation) were measured among the subjects with different genotypes of the *FABP1* rs2919872G>A (GG genotype: 13.67 ± 2.60 ng/mL (n = 53); GA genotype: 9.44 ± 2.29 ng/mL (n = 32); AA genotype: 5.13 ± 4.38 ng/mL (n = 10); *P < 0.01)

Serum FABP1 levels correlate closely with those in the liver, which is main site of FABP1 expression [15]. To investigate the association between the rs2919872 genotype and serum FABP1 levels in our study population, FABP1 levels were evaluated by ELISA in serum samples from 95 individuals with different rs2919872A or rs2919872G alleles. There were no significant differences in the distributions of age, sex, marital status, education or income among the different rs2919872 genotypes (all *P* > 0.05). As shown in Fig 3, subjects with the rs2919872 AA genotype had significantly lower serum FABP1 concentrations (geometric mean ± geometric standard deviation) than those carrying the GG genotypes (GG genotype: 13.67 ± 2.60 ng/mL, *n* = 53 versus AA genotype: 5.13 ± 4.38 ng/mL, *n* = 10, *P* < 0.01). Multiple linear regression analysis to predict the variables independently associated with serum FABP1 showed that, after adjustment for all the covariables in Table 4, rs2919872 A allele was negatively (β = -0.331, *P* = 0.003) associated with serum logFABP1 concentrations, while serum TG levels were positively (β = 0.487, *P* = 0.014) associated with serum logFABP1 concentrations(Table 4). These results suggested that rs2919872 is associated with TG levels by modulating *FABP1* expression.

**Table 4:** Variables independently associated with plasma FABP1 levels by multivariate lineal regression analysis.

Variables	Standardized coefficient(*β*)	*P*	Adjusted R^2^
			0.250
rs2919872 G>A (recessive model)	-0.320	0.003	
TG	0.487	0.014	
ALT	0.045	0.837	
AST	-0.018	0.926	
SABP	0.140	0.511	
DABP	-0.136	0.512	
HDL-C	0.059	0.761	
LDL-C	0.680	0.151	
TC	0.066	0.209	
BMI	-0.046	0.746	
Age	-0.118	0.349	
Sex	0.230	0.051	

All variables were entered into this model in a single step.

## Discussion

In this study, we investigated the common polymorphisms located in the promoter region of the FABP1 gene that may affect lipid metabolism by influencing *FABP1* gene transcription. To the best of our knowledge, this is the first study to examine the association of *FABP1* promoter polymorphisms with lipid levels. In this study, decreased levels of serum *FABP1* were observed in subjects with the rs2919872 A allele, indicating that a G>A transition at rs2919872, which is located in the promoter region of the *FABP1* gene is responsible for a major effect on serum TG levels in a Chinese Han population. Furthermore, *in vitro* experiments indicated that the *FABP1* rs2919872 A allele decreased the transcriptional activity of the *FABP1* gene, probably due to the disturbance of a transcription factor binding site, suggesting the functional relevance of this site.

FABP1 is a key regulator of hepatic lipid metabolism and is required for optimal activity of transacylase enzymes in the murine TG synthesis pathway. Overexpression of murine FABP1 is associated with increased LCFA uptake and hepatic TG levels [25,26,27], while *FABP1* ^−/−^ mice exhibit decreased hepatic triglyceride content with altered FA uptake kinetics [12], with protection against diet-induced obesity and hepatic steatosis [14,28]. In humans, amino acid variations in *FABP1* could be functionally relevant. The functional mutation rs2241883 (p.Thr94Ala) in the *FABP1* gene has been extensively studied, and the Ala94/Ala polymorphism shows a significant association with increased serum LDL-C and TG levels[15], as well as decreased response to lipid-lowering therapy with fenofibrate (a cholesterol synthesis inhibitor) and glycogenolysis [16,29,30]. To date, little is known about the potential influence of nucleic acid variants in the *FABP1* gene promoter on lipid metabolism. In the present study, we analyzed four polymorphisms that appeared to be relevant in the regulation of FABP1 expression. We found that only the rs2919872 A allele contributed significantly to reduced serum TG levels in a Chinese population. Furthermore, regression analyses also showed a similar independent inverse association of the *FABP1* rs2919872 G>A variant with TG levels and that, together with the BMI, the concentrations of TC, HDL-C, LDL-C, SBP, and ALT, explained approximately 30% of TG variability. The increased minor allele frequency in populations with lower TG levels suggests that the A allele of the rs2919872 of the *FABP1* gene may influence *FABP1* expression and protect against hypertriglyceridemia. This hypothesis is further supported by physiological data as well as by genetic findings. Functional assays using the luciferase gene as a reporter that were performed as part of this study showed that the A allele almost abolished the promoter activity of *FABP1* gene compared with effect of the G allele. In addition, we also found that healthy individuals carrying with the A allele had a considerably lower serum FABP1 concentration than the G allele carriers. Furthermore, regression analyses showed that rs2919872 G>A was negatively associated with serum FABP1 concentrations, while serum TG levels were positively associated with serum FABP1 concentrations after controlling for potential confounding factors. It was noteworthy that eQTL data analysis showed that only one SNP (rs2241883) was reported to be associated with *FABP1* mRNA levels, however, this SNP was not in high LD with rs2919872.The observed phenotype shows obvious conformity with that of *FABP1* knockout mice [12,14,28], indicating that the rs2919872 G>A replacement in *FABP1* is likely to be a ‘loss-of-function’ variant analogous to the *FABP1* null mouse. It is noteworthy that our findings are also consistent with those obtained for the rs2241883 (p.Thr94Ala) polymorphism. The FABP1 T94A substitution in this variant was shown to be associated with upregulation of total FABP1, which in turn may stimulate enzymes in the TG synthesis pathway and lead to the elevated plasma TGs in human subjects [15,31].

The association of downregulated FABP1 with protection against high TG may be explained in two ways. First, because FABP1 is involved in the budding of pre-chylomicron transport vesicles from the endoplasmic reticulum of the enterocyte [32], downregulation of *FABP1* would be expected to result in subtle differences in intestinal FA trafficking and delayed appearance of dietary TG in the serum [12,14]. Second, FABP1 is abundantly expressed in both hepatocytes and enterocytes and binds to multiple ligands, including saturated FA and cholesterol[12]; therefore downregulation of *FABP1* would result in impaired FA uptake, decreased production of very low density lipoproteins, and reduced hepatic TG accumulation.

The mechanisms involved in the downregulation of FABP1 by the G-to-A substitution at rs2919872 in the promoter region remain to be elucidated. However, it can be speculated that the rs2919872 G>A mutation either generates a binding site for a negative regulator or abolishes the existing binding site for a positive regulator. However, besides the MatInspector and CHIP Mapper, searches of the TESS (http://www.cbil.upenn.edu/cgi-bin/tess/tess) and TFSEARCH (http://www.cbrc.jp/research/db/TFSEARCH.html) databases did not reveal any potential binding sites changes for transcription factors due to the presence of rs2919872 A(data not shown). Therefore, further DNA pull-down and yeast one hybrid screening studies are required to identify the possible function of rs2919872 G>A.

Several limitations in our study need to be discussed. Firstly, in the present study, although the rs2919872 variant affected transcription efficiency of the *FABP1*’s promoter and the levels of FABP1 protein, *FABP1* mRNA levels for each SNP allele or genotype were not measured in our population. However, this limitation is unavoidable, because for ethical reasons it is hard for us to obtain liver tissue even the biopsy samples from individuals taking a regular medical health check-up. Secondly, the SNPs were chosen to maximize SNP tagging for genetic variation rather than for the functionality of the *FABP1* gene in the present study. Although the luciferase data indicated that only rs2919872 SNP was functional, it did not exclude the possibility that other SNPs in high LD may be responsible for the observed associations, the exact mechanisms linking rs2919872 A allele to lower serum TG levels in humans needs further studies. Thirdly, the weak *P* value of association in the initial population (*P* = 0.032) was not corrected for multiple testing. Multiple testing correction is necessary to exclude false-positive loci, however, it may simultaneously hid many true-positive loci which were biologically associated with clinical traits[20,21]. Therefore, these reports suggested that reducing the number of tests may be advantageous for finding true-positive loci [21,22]. Finally, our study firstly reported the relationship between SNPs (rs2919872) in *FABP1* gene and serum TG levels in Chinese Han subjects. It is unclear whether the present findings can be generalized to other ethnicities. Thus, only specific testing of putative causal variants directly in extensive cohorts and population-based samples will help validate the role of *FABP1* rs2919872 in lipid metabolism.

In summary, the results of this study demonstrate that rs2919872 G>A, a common polymorphism in the promoter region of *FABP1*, may be of functional importance in regulating the expression of the *FABP1* gene and might influence the serum TG concentrations in a Chinese Han population. Considering the association of increased serum TG levels with increased risk of metabolic syndrome and CVD [2,33,34], this variant still represents an interesting potential target for future lipid-lowering therapies to reduce the risk of CVD.

## Abbreviations

ALT: alanine aminotransferase
AST: aspartate aminotransferase
BMI: body mass index
CVD: cardiovascular disease
DABP: diastolic arterial blood pressure
FABP1: fatty acid-binding protein 1
FPG: fasting plasma glucose
HWE: Hardy–Weinberg Equilibrium
HDL-C: high density lipoprotein cholesterol
LD: linkage disequilibrium
LDL-C: low density lipoprotein cholesterol
L-FABP: Liver fatty acid-binding protein
NAFLD: nonalcoholic fatty liver disease
SABP: systolic arterial blood pressure
SNP: single-nucleotide polymorphisms
TC: total cholesterol
TG: triglyceride

## References

[1] Degoma EM, Rader DJ (2011) Novel HDL-directed pharmacotherapeutic strategies. Nat Rev Cardiol 8: 266–277. 10.1038/nrcardio.2010.200 21243009 PMC3315102

[2] Nordestgaard BG, Varbo A (2014) Triglycerides and cardiovascular disease. Lancet 384: 626–635. 10.1016/S0140-6736(14)61177-6 25131982

[3] Eeg-Olofsson K, Gudbjornsdottir S, Eliasson B, Zethelius B, Cederholm J (2014) The triglycerides-to-HDL-cholesterol ratio and cardiovascular disease risk in obese patients with type 2 diabetes: an observational study from the Swedish National Diabetes Register (NDR). Diabetes Res Clin Pract 106: 136–144. 10.1016/j.diabres.2014.07.010 25108897

[4] Miller M, Stone NJ, Ballantyne C, Bittner V, Criqui MH, et al (2011) Triglycerides and cardiovascular disease: a scientific statement from the American Heart Association. Circulation 123: 2292–2333. 10.1161/CIR.0b013e3182160726 21502576

[5] Zhang Y, Smith EM, Baye TM, Eckert JV, Abraham LJ, et al (2010) Serotonin (5-HT) receptor 5A sequence variants affect human plasma triglyceride levels. Physiol Genomics 42: 168–176. 10.1152/physiolgenomics.00038.2010 20388841 PMC3032280

[6] Kathiresan S, Willer CJ, Peloso GM, Demissie S, Musunuru K, et al (2009) Common variants at 30 loci contribute to polygenic dyslipidemia. Nat Genet 41: 56–65. 10.1038/ng.291 19060906 PMC2881676

[7] Willer CJ, Sanna S, Jackson AU, Scuteri A, Bonnycastle LL, et al (2008) Newly identified loci that influence lipid concentrations and risk of coronary artery disease. Nat Genet 40: 161–169. 10.1038/ng.76 18193043 PMC5206900

[8] Bass NM, Manning JA (1986) Tissue expression of three structurally different fatty acid binding proteins from rat heart muscle, liver, and intestine. Biochem Biophys Res Commun 137: 929–935. 3729957 10.1016/0006-291x(86)90314-1

[9] Pelsers MM, Namiot Z, Kisielewski W, Namiot A, Januszkiewicz M, et al (2003) Intestinal-type and liver-type fatty acid-binding protein in the intestine. Tissue distribution and clinical utility. Clin Biochem 36: 529–535. 14563446 10.1016/s0009-9120(03)00096-1

[10] Petrescu AD, McIntosh AL, Storey SM, Huang H, Martin GG, et al (2013) High glucose potentiates L-FABP mediated fibrate induction of PPARalpha in mouse hepatocytes. Biochim Biophys Acta 1831: 1412–1425. 10.1016/j.bbalip.2013.05.008 23747828 PMC3730521

[11] Veerkamp JH, van Moerkerk HT (1993) Fatty acid-binding protein and its relation to fatty acid oxidation. Mol Cell Biochem 123: 101–106. 8232250 10.1007/BF01076480

[12] Newberry EP, Xie Y, Kennedy S, Han X, Buhman KK, et al (2003) Decreased hepatic triglyceride accumulation and altered fatty acid uptake in mice with deletion of the liver fatty acid-binding protein gene. J Biol Chem 278: 51664–51672. 14534295 10.1074/jbc.M309377200

[13] Chen A, Tang Y, Davis V, Hsu FF, Kennedy SM, et al (2013) Liver fatty acid binding protein (L-Fabp) modulates murine stellate cell activation and diet-induced nonalcoholic fatty liver disease. Hepatology 57: 2202–2212. 10.1002/hep.26318 23401290 PMC3665693

[14] Newberry EP, Xie Y, Kennedy SM, Luo J, Davidson NO (2006) Protection against Western diet-induced obesity and hepatic steatosis in liver fatty acid-binding protein knockout mice. Hepatology 44: 1191–1205. 17058218 10.1002/hep.21369

[15] Fisher E, Weikert C, Klapper M, Lindner I, Mohlig M, et al (2007) L-FABP T94A is associated with fasting triglycerides and LDL-cholesterol in women. Mol Genet Metab 91: 278–284. 17485234 10.1016/j.ymgme.2007.03.002

[16] Brouillette C, Bosse Y, Perusse L, Gaudet D, Vohl MC (2004) Effect of liver fatty acid binding protein (FABP) T94A missense mutation on plasma lipoprotein responsiveness to treatment with fenofibrate. J Hum Genet 49: 424–432. 15249972 10.1007/s10038-004-0171-2

[17] Peng XE, Wu YL, Lu QQ, Hu ZJ, Lin X (2012) Two genetic variants in FABP1 and susceptibility to non-alcohol fatty liver disease in a Chinese population. Gene 500: 54–58. 10.1016/j.gene.2012.03.050 22465531

[18] Riva A (2012) The MAPPER2 Database: a multi-genome catalog of putative transcription factor binding sites. Nucleic Acids Res 40: D155–161. 10.1093/nar/gkr1080 22121218 PMC3245066

[19] Wu YL, Peng XE, Wang D, Chen WN, Lin X (2012) Human liver fatty acid binding protein (hFABP1) gene is regulated by liver-enriched transcription factors HNF3beta and C/EBPalpha. Biochimie 94: 384–392. 10.1016/j.biochi.2011.08.006 21856370

[20] Fransen K, Visschedijk MC, van Sommeren S, Fu JY, Franke L, et al (2010) Analysis of SNPs with an effect on gene expression identifies UBE2L3 and BCL3 as potential new risk genes for Crohn's disease. Hum Mol Genet 19: 3482–3488. 10.1093/hmg/ddq264 20601676

[21] Hong KW, Jin HS, Lim JE, Cho YS, Go MJ, et al (2010) Non-synonymous single-nucleotide polymorphisms associated with blood pressure and hypertension. J Hum Hypertens 24: 763–774. 10.1038/jhh.2010.9 20147969

[22] Hong KW, Lim JE, Oh B (2011) A regulatory SNP in AKAP13 is associated with blood pressure in Koreans. J Hum Genet 56: 205–210. 10.1038/jhg.2010.167 21228793

[23] Guzman C, Benet M, Pisonero-Vaquero S, Moya M, Garcia-Mediavilla MV, et al (2013) The human liver fatty acid binding protein (FABP1) gene is activated by FOXA1 and PPARalpha; and repressed by C/EBPalpha: Implications in FABP1 down-regulation in nonalcoholic fatty liver disease. Biochim Biophys Acta 1831: 803–818. 10.1016/j.bbalip.2012.12.014 23318274

[24] Brahe LK, Angquist L, Larsen LH, Vimaleswaran KS, Hager J, et al (2013) Influence of SNPs in nutrient-sensitive candidate genes and gene-diet interactions on blood lipids: the DiOGenes study. Br J Nutr 110: 790–796. 10.1017/S0007114512006058 23360819

[25] Murphy EJ, Prows DR, Jefferson JR, Schroeder F (1996) Liver fatty acid-binding protein expression in transfected fibroblasts stimulates fatty acid uptake and metabolism. Biochim Biophys Acta 1301: 191–198. 8664328 10.1016/0005-2760(96)00024-0

[26] Murphy EJ (1998) L-FABP and I-FABP expression increase NBD-stearate uptake and cytoplasmic diffusion in L cells. Am J Physiol 275: G244–249. 9688651 10.1152/ajpgi.1998.275.2.G244

[27] Wolfrum C, Buhlmann C, Rolf B, Borchers T, Spener F (1999) Variation of liver-type fatty acid binding protein content in the human hepatoma cell line HepG2 by peroxisome proliferators and antisense RNA affects the rate of fatty acid uptake. Biochim Biophys Acta 1437: 194–201. 10064902 10.1016/s1388-1981(99)00008-6

[28] Newberry EP, Kennedy SM, Xie Y, Luo J, Davidson NO (2009) Diet-induced alterations in intestinal and extrahepatic lipid metabolism in liver fatty acid binding protein knockout mice. Mol Cell Biochem 326: 79–86. 10.1007/s11010-008-0002-4 19116776 PMC3004673

[29] Weickert MO, Loeffelholz CV, Roden M, Chandramouli V, Brehm A, et al (2007) A Thr94Ala mutation in human liver fatty acid-binding protein contributes to reduced hepatic glycogenolysis and blunted elevation of plasma glucose levels in lipid-exposed subjects. Am J Physiol Endocrinol Metab 293: E1078–1084. 17698986 10.1152/ajpendo.00337.2007

[30] Baier LJ, Sacchettini JC, Knowler WC, Eads J, Paolisso G, et al (1995) An amino acid substitution in the human intestinal fatty acid binding protein is associated with increased fatty acid binding, increased fat oxidation, and insulin resistance. J Clin Invest 95: 1281–1287. 7883976 10.1172/JCI117778 PMC441467

[31] McIntosh AL, Huang H, Storey SM, Landrock KK, Landrock D, et al (2014) Human FABP1 T94A variant impacts fatty acid metabolism and PPAR-alpha activation in cultured human female hepatocytes. Am J Physiol Gastrointest Liver Physiol 307: G164–176. 10.1152/ajpgi.00369.2013 24875102 PMC4101680

[32] Neeli I, Siddiqi SA, Siddiqi S, Mahan J, Lagakos WS, et al (2007) Liver fatty acid-binding protein initiates budding of pre-chylomicron transport vesicles from intestinal endoplasmic reticulum. J Biol Chem 282: 17974–17984. 17449472 10.1074/jbc.M610765200

[33] Boullart AC, de Graaf J, Stalenhoef AF (2012) Serum triglycerides and risk of cardiovascular disease. Biochim Biophys Acta 1821: 867–875. 10.1016/j.bbalip.2011.10.002 22015388

[34] Chen BD, Yang YN, Ma YT, Pan S, He CH, et al. (2015) Waist-to-Height Ratio and Triglycerides/High-Density Lipoprotein Cholesterol Were the Optimal Predictors of Metabolic Syndrome in Uighur Men and Women in Xinjiang, China. Metab Syndr Relat Disord. 10.1089/met.2014.0146 25781351

